# Immuno-therapeutic and prophylactic potential of *Trichinella spiralis* antigens for inflammatory bowel diseases

**DOI:** 10.1016/j.fawpar.2024.e00248

**Published:** 2024-10-06

**Authors:** Majed H. Wakid, Walaa A. El Kholy, Muslimah N. Alsulami, Eman S. El-Wakil

**Affiliations:** a Department of Medical Laboratory Sciences, Faculty of Applied Medical Sciences, King Abdulaziz University, Jeddah, Saudi Arabia; b Special Infectious Agents Unit, King Fahd Medical Research Center, King Abdulaziz University, Jeddah, Saudi Arabia; c Department of Parasitology, Faculty of Medicine for Girls, Al-Azhar University, Cairo, Egypt; d Department of Biology, College of Science, University of Jeddah, Jeddah, Saudi Arabia; e Department of Parasitology, Theodor Bilharz Research Institute, Kornaish El-Nile, Warrak El-Hadar, Imbaba, Giza, Egypt

**Keywords:** Trichinella spiralis, Inflammatory bowel diseases, Adult antigens, Larval antigens, Acetic acid-induced colitis

## Abstract

Ulcerative colitis (UC), a severe chronic inflammatory disorder of the colon, is one of the inflammatory bowel diseases (IBD) that affects humans and several domestic animal species, including cats and dogs. Helminth infections and autoimmune diseases are inversely correlated, as explained by the hygiene hypothesis, which suggests that IBD is infrequent in countries where helminth infections are common but more prevalent in developed nations. This study investigated the therapeutic and prophylactic potential of *Trichinella spiralis* (*T. spiralis*) antigens in an experimental colitis model for IBD. Mice were divided into eight groups: normal model, colitis model, larval antigen prophylaxis, adult antigen prophylaxis, larval antigen therapeutic, adult antigen therapeutic, larval antigen prophylaxis and therapeutic, and adult antigen prophylaxis and therapeutic. Colitis was induced intrarectally by administering a single dose of 0.2 ml of acetic acid, except in the healthy group, which received PBS (0.2 ml). The mice were euthanized 12 days after colitis induction. The therapeutic and prophylactic potential of *T. spiralis* antigens were assessed through colitis severity and histopathological, immunological, and immunohistochemical examinations. The results showed a significant reduction in Disease Activity Index (DAI), an increase in goblet cells' acidic mucin levels, reduced iNOS and TNF-α expression, and decreased serum levels of IFN-γ and IL-10 cytokines in Groups IV-VIII compared to the colitis model, particularly in the group that received adult worm antigen both prophylactically and therapeutically. This study demonstrated that *T. spiralis* antigens, especially from adult worms, had protective and therapeutic effects on experimental colitis, with a superior effect when administered both before and after colitis induction by reducing inflammation and modulating the immune response. Thus, *T. spiralis* antigens may improve disease outcomes and provide a novel treatment approach for ulcerative colitis.

## 1 Introduction

The ongoing rise in inflammatory bowel disease (IBD) cases poses a global health threat. In addition to humans, IBD can affect the gastrointestinal tracts of several domestic animals, including cats and dogs (Allenspach et al., 2019; Meineri et al., 2022; Pratscher et al., 2024). IBDs are classified into ulcerative colitis (UC) and Crohn's disease (CD). UC is a chronic inflammatory condition affecting the colon and rectum, characterized by bloody diarrhea and abdominal pain, with the potential to cause colitis-related cancer if treatment is ineffective (Saber et al., 2021; Shaaban et al., 2022). Although the pathogenesis and etiology of these disorders remain unclear, it is believed that an intricate interaction between genetic, microbial, and environmental factors is responsible for the abnormal immune responses that lead to intestinal inflammation (Yang et al., 2014).

Treatment for UC can involve medication or surgery, depending on the severity of the illness and the extent of involvement. Medications for UC treatment include corticosteroids, 5-aminosalicylic acid, biologics, and immunomodulators. Surgery is an option for patients with intestinal perforation or highly suspected malignancy. However, both long-term medication use and surgery carry potential side effects (Cosnes et al., 2011). A significant improvement over current treatments would be the development of a medication that could restore immune system balance and target the mechanisms underlying the disease's development and progression (Kotla and Rochev, 2023).

Helminths and humans have coevolved, and it is believed that these parasites play a role in maintaining the balanced immune response necessary for immune regulation (Smallwood et al., 2017). The ability of helminths to protect against inflammatory diseases is supported by the hygiene hypothesis, which links the increase in allergic and autoimmune inflammatory diseases to reduced exposure to gastrointestinal helminths and commensal microorganisms in developed countries (Zhang and Gems, 2021). Many autoimmune diseases have long been treated with helminthic infections, and extensive epidemiological, experimental, and clinical evidence suggests that these infections modulate the immune system, potentially strengthening defenses against autoimmune disorders (Bach, 2018).

However, using live infections raises safety and ethical concerns, leading researchers to explore the potential of helminth-derived products to improve autoimmune diseases and develop new treatment methods for inflammatory disorders (Sotillo et al., 2017). Helminthic infections have shown positive effects on the course of several autoimmune diseases, including IBD (Elliott and Weinstock, 2012). Given the risks associated with live helminthic therapy, research has focused on identifying helminth-derived compounds that can modulate the immune system and safely administered to patients (Harnett and Harnett, 2010). Thus, using helminth-derived antigens, as investigated in this research, holds promise for mitigating the effects of several autoimmune and allergic conditions, including airway disease, Type 1 diabetes, experimental autoimmune encephalomyelitis, and IBD (Eissa et al., 2016).

One of the common zoonotic parasitic nematodes is *Trichinella spiralis*. Its life cycle is completed within a single host and consists of three stages: adult worms in the small intestine, newborn larvae in the circulation, and muscle larvae inside skeletal striated muscle cells. *T. spiralis* releases several proteins into its surrounding environment at different stages of its development, which are believed to be essential for its successful invasion and survival within the host. These proteins help *T. spiralis* evade or inhibit the host immune response (Yang et al., 2014).

Growing experimental evidence has highlighted *T. spiralis* infection as a potential treatment for several autoimmune and allergic disorders, including autoimmune encephalitis, airway disease, Type 1 diabetes, and inflammatory bowel disease (Eissa et al., 2016). The use of animals remains central to preclinical drug development. Significant progress in understanding IBD's etiology, management, and prognosis has been made possible by developing experimental techniques using animal models (Lee et al., 2023). The present study investigated the therapeutic and prophylactic potential of *T. spiralis* antigens in an experimental colitis model for IBD.

## 2 Materials and methods

### 2.1 Ethics statement

All laboratory animal procedures were conducted following internationally recognized guidelines and approved by the Research and Ethics Committee of the Faculty of Applied Medical Sciences at King Abdulaziz University, KSA (Approval No. FAMS-EC2022–08).

### 2.2 Parasites

In this study, *T. spiralis* ISS6158 was used, maintained at the Parasitology Department, Theodor Bilharz Research Institute (TBRI), through consecutive passages in mice (Gamble, 1996). *T. spiralis* adults and muscle larvae from infected mice were recovered according to previous studies (Ozkoc et al., 2009). Briefly, adult *T. spiralis* worms were extracted from the small intestine of infected mice five days post-infection (DPI). The intestines were cleaned, cut into 2 cm sections, and opened lengthwise. They were submerged in normal saline for 3–4 h at 37 °C to allow the worms to exit the tissue. Infected muscles from mice were minced and digested with 1 % pepsin-HCL solution at 30 DPI. After overnight incubation at 37 °C, larvae were collected using the sedimentation technique and repeatedly rinsed with normal saline.

### 2.3 Parasitic antigen preparation

Adult *T. spiralis* worms and muscle larvae were used to prepare antigens, according to Escalante et al. (2004). Briefly, adult worms or muscle larvae were resuspended in phosphate-buffered saline (PBS), homogenized, and centrifuged (3000 x*g* for 30 min at 4 °C). After discarding the pellets, the supernatants were further centrifuged at 40,000 x*g* for 60 min at 4 °C. The antigen-containing supernatants were collected, lyophilized, and stored at −20 °C until use (Eissa et al., 2016). The protein content of both antigens was quantified using a NanoDrop™ 2000 spectrophotometer.

### 2.4 Animals and experimental design

**Fig. 1:**
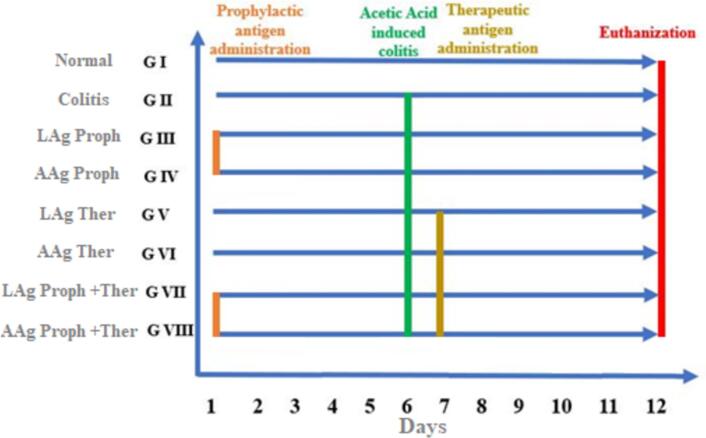
Schematic illustration of the timeline for antigens administration and colitis induction.

The study was conducted on 48 laboratory-bred male white albino mice of the CDI strain (six to eight weeks old, weighing 25–30 g). The animals were housed in sterile conditions (20–24 °C, 12-h light/dark cycle) with unrestricted access to sterilized food and water. Mice were randomly assigned to eight groups (six mice per group) (Fig. 1):

- Group I (Normal): Healthy mice.
- Group II (Colitis model): Mice administered acetic acid.
- Group III (LAg Proph.): Mice received muscle larvae antigen intraperitoneally (100 μg) for five days before acetic acid administration (prophylactic).
- Group IV (AAg Proph.): Mice received adult worm antigen intraperitoneally (100 μg) for five days before acetic acid administration (prophylactic).
- Group V (LAg Ther.): Mice received muscle larvae antigen intraperitoneally (100 μg) for five days after acetic acid administration (therapeutic) (Motomura et al., 2009).
- Group VI (AAg Ther.): Mice received adult worm antigen intraperitoneally (100 μg) for five days after acetic acid administration (therapeutic).
- Group VII (LAg Proph. + Ther.): Mice received muscle larvae antigen intraperitoneally (100 μg) for five days before (prophylactic) and five days after acetic acid administration (therapeutic).
- Group VIII (AAg Proph. + Ther.): Mice received adult worm antigen intraperitoneally (100 μg) for five days before (prophylactic) and five days after acetic acid administration (therapeutic) (Motomura et al., 2009).

### 2.5 Induction of colitis

Colitis was induced by administering a single dose of 0.2 ml of acetic acid (5 %, pH 2.5) intrarectally, 3 cm from the anal margin. For the healthy group, mice received 0.2 ml of PBS (Ghasemi-Dehnoo et al., 2022).

### 2.6 Assessment of the degree of colitis

#### 2.6.1 Weight-to-length ratio of colon

A 10 cm section of the distal colon was resected, opened lengthwise, and flushed with normal saline to remove feces. The colon weight-to-length ratios were measured blindly, with the colons positioned on non-absorbent surfaces (Shahid et al., 2022).

#### 2.6.2 Disease activity index (DAI)

For colitis assessment, body weight loss, stool consistency, and fecal bleeding were analyzed on a zero to four-point scale and averaged to calculate the overall DAI (See Table 1), (Saber and El-Kader, 2021).

**Table 1:** Parameters of Disease Activity Index scoring.

Score	Body weight loss%	Stool consistency	Bleeding
0	Less than 1 %	Normal	Normal
1	1–5 %	Loose	Slight
2	6–10 %	Loose	Slight
3	11–20 %	Diarrhea	Gross
4	More than 20 %	Diarrhea	Gross

### 2.7 Histopathological examination

The animals were sacrificed on the 12th day of the experiment under light anesthesia. Standard procedures for fixation and staining were performed following the method of Culling (2013). Additionally, an Alcian blue stain (pH = 2.5) was used to visualize the acidic mucins in goblet cells. At least six non-overlapping fields were randomly selected and scanned from each sample to determine the mucosal area percentage of reactive goblet cells' mucin content (Khedr et al., 2023).

### 2.8 Immunohistochemical assessment of inducible nitric oxide synthase (iNOS) and tumor necrosis factor alpha (TNF-α) expressions

Paraffin-embedded colonic tissue sections (five μm thick) were prepared according to the manufacturer's protocol for immunohistochemical analysis. The deparaffinized tissue sections were treated with 0.3 % H_2_O_2_ for 20 min. Samples were then incubated overnight with Anti-iNOS (1:50 dilution) (MA5–16422, Thermo Fisher Scientific Co., Waltham, Massachusetts, USA) and Anti-TNF-α (1:100 dilution) (NBP1–19532, Novus Bio. Co., Easter Ave Centennial, USA) at 4 °C. The tissue sections were washed with PBS and incubated with the secondary antibody HRP Envision kit (DAKO, Santa Clara, CA, USA) for 20 min, followed by incubation with diaminobenzidine (DAB) for 15 min. After washing with PBS, the sections were counterstained with hematoxylin, dehydrated, cleared in xylene, and cover-slipped for microscopic examination.

For histological analysis, at least six non-overlapping fields were randomly selected from each immunohistochemically stained section to determine the mean reactive iNOS and TNF-α cell counts per microscopic field (Khedr et al., 2023). A Leica application unit for histological analysis, connected to a high-resolution (full HD) microscopic imaging system, was used to collect all light microscopy data (Leica Microsystems GmbH, Germany).

### 2.9 Immunological analysis of serum levels of interferon gamma (IFN-γ) and interleukin-10 (IL-10) cytokines

Blood samples were centrifuged for five minutes at 1811 x*g*, and the obtained serum was stored at −20 °C until analysis. IFN-γ and IL-10 levels were measured according to the manufacturer's instructions for ELISA assays (FineTest, Wuhan, China, and Elabscience, Texas, USA, respectively).

### 2.10 Statistical analysis

The Statistical Package for the Social Sciences (IBM SPSS Statistics for Windows, Version 26, IBM Corp., Armonk, NY, USA) was used for data analysis. Normally distributed continuous variables were presented as mean ± SE. For multigroup comparisons, one-way ANOVA followed by Tukey's HSD post hoc test was used. A *p*-value <0.05 was considered statistically significant, and < 0.01 was considered highly significant.

## 3 Results

### 3.1 The degree of colitis

**Fig. 2:**
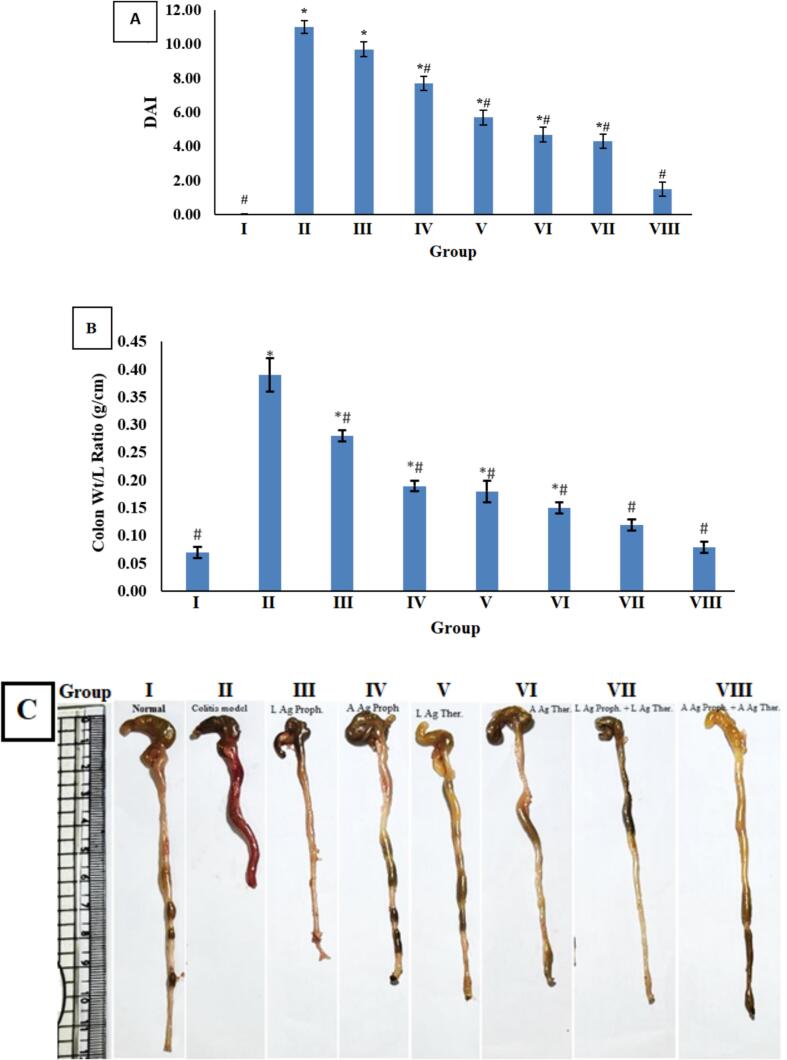
A: Disease activity index; B: Weight-to-Length Ratio of Colon (data are displayed as mean ± SE). *p < 0.05 versus normal model (Group I), *#p* < 0.05 versus colitis model (Group II). C: Photomicrograph of harvested colons from all experimental groups.

As shown in Table 2 and Fig. 2, the colitis model (Group II) exhibited a significant increase in the colon weight/length ratio and DAI, demonstrated by decreased body weight, diarrhea, and severe bloody stools, compared to the healthy mice in Group I. The prophylactic administration of *T. spiralis* muscle larval antigens to mice before acetic acid-induced colitis (Group III) showed no significant difference (*p* < 0.05) in DAI compared to the colitis model (Group II). However, a highly significant decrease (*p* < 0.01) in DAI was observed in Groups IV–VIII. A marked decrease in DAI, with no significant difference (p < 0.05) from the healthy mice (Group I), was indicated by the improvement in body weight and the resolution of colitis clinical symptoms in Group VIII, which received *T. spiralis* adult antigens both before and after acetic acid administration.

**Table 2:** Disease Activity Index scoring in different experimental groups.

Group		I	II	III	IV	V	VI	VII	VIII
Mean ± SE		0.05 ± 0.02	11 ± 0.37	9.7 ± 0.42	7.7 ± 0.4	5.7 ± 0.42	4.7 ± 0.4	4.3 ± 0.42	1.5 ± 0.43
*p*-value ^a^ = 0.001									
***p*-value**^**b**^	I	–	0.001	0.001	0.001	0.001	0.001	0.001	0.2
	II	–	–	0.3	0.001	0.001	0.001	0.001	0.001
	III	–	–	–	0.02	0.001	0.001	0.001	0.001
	IV	–	–	–	–	0.02	0.001	0.001	0.001
	V	–	–	–	–	–	0.6	0.3	0.001
	VI	–	–	–	–	–	–	1	0.001
	VII	–	–	–	–	–	–	–	0.001

DAI scoring is represented as the mean ± SE. *P*-value ^a^ indicates a significant difference according to one-way ANOVA when comparing groups. *P*-value ^b^ indicates a significant difference in comparing groups according to the Tukey HSD post hoc test. A *p*-value <0.05 is considered significant, and < 0.01 is considered highly significant.

### 3.2 Histopathological findings

#### 3.2.1 Hematoxylin and eosin stain findings

**Fig. 3:**
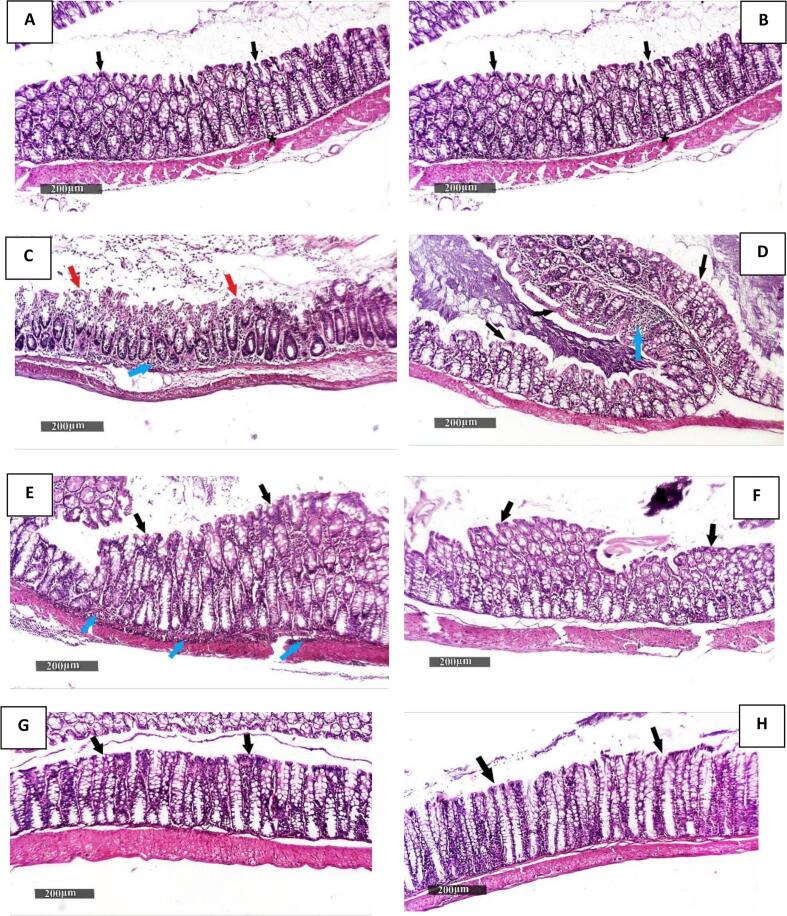
Photomicrograph of colon tissue sections (stained with H&E). **A**: Group I exhibit a normal colon wall. **B**: Group II revealed marked mucosal ulceration and necrotic tissue debris (red arrow) with significant infiltration of mixed severe inflammatory cells (blue arrow). **C**: Group III shows minimal protective efficacy, presenting almost the same histological characteristics as the model samples. **D**: Group IV displays significant improvement in colonic wall histological organization. **E**: Group V shows significant improvement in colonic wall histological organization and a significant increase in mature goblet cells (black arrow) alongside inflammatory cells (blue arrow). **F**: Group VI demonstrates well-organized histology of the colonic wall with proven protective efficacy (black arrow). **G**: Group VII displays well-organized histological characteristics of the colonic wall with proven protective efficacy (black arrow). **H**: Group VIII shows the best-organized morphological features among the treated/protected groups, resembling normal controls (black arrow). (For interpretation of the references to colour in this figure legend, the reader is referred to the web version of this article.)

Microscopic examination of colon samples from Group I (normal control) revealed typical colon wall histology, including intact intestinal crypts with numerous goblet cells and an intact outer layer epithelium, along with a normal submucosa and outer muscular coat (Fig. 3A). Group II (colitis model) samples exhibited marked mucosal ulceration and necrotic tissue debris, with significant disorganization of colonic wall morphology, mixed severe inflammatory cell infiltration, and moderate submucosal and intermuscular edema (Fig. 3B). The third group (LAg Proph.) demonstrated minimal protective efficacy, showing approximately the same histological characteristics as the model samples (Fig. 3C). Group IV (AAg Proph.) showed significant improvement in the histological organization of the colon wall, with clearly intact lining epithelium and mucosal glandular structures and a significant increase in mature goblet cells. However, occasional focal aggregates of inflammatory mononuclear cells were observed (Fig. 3D). Group V (LAg Ther.) exhibited significant improvement in the histological organization of the colon wall, with apparent intact lining epithelium and mucosal glandular structures, alongside a significant increase in mature goblet cells.

Additionally, intense sheets of mucosal/submucosal inflammatory cells were detected in most of the samples (Fig. 3E). Group VI (AAg Ther.) displayed a well-organized colonic wall with proven protective efficacy (Fig. 3F). Group VII (LAg Proph. + Ther.) showed a well-organized colonic wall with proven protective efficacy, although occasional records of minor mucosal aggregates of inflammatory cells were noted (Fig. 3G). Group VIII (AAg Proph. + Ther.) exhibited the best-organized morphological features among the treated/protected groups, resembling normal controls (Fig. 3H).

#### 3.2.2 Alcian blue stain findings

**Fig. 4:**
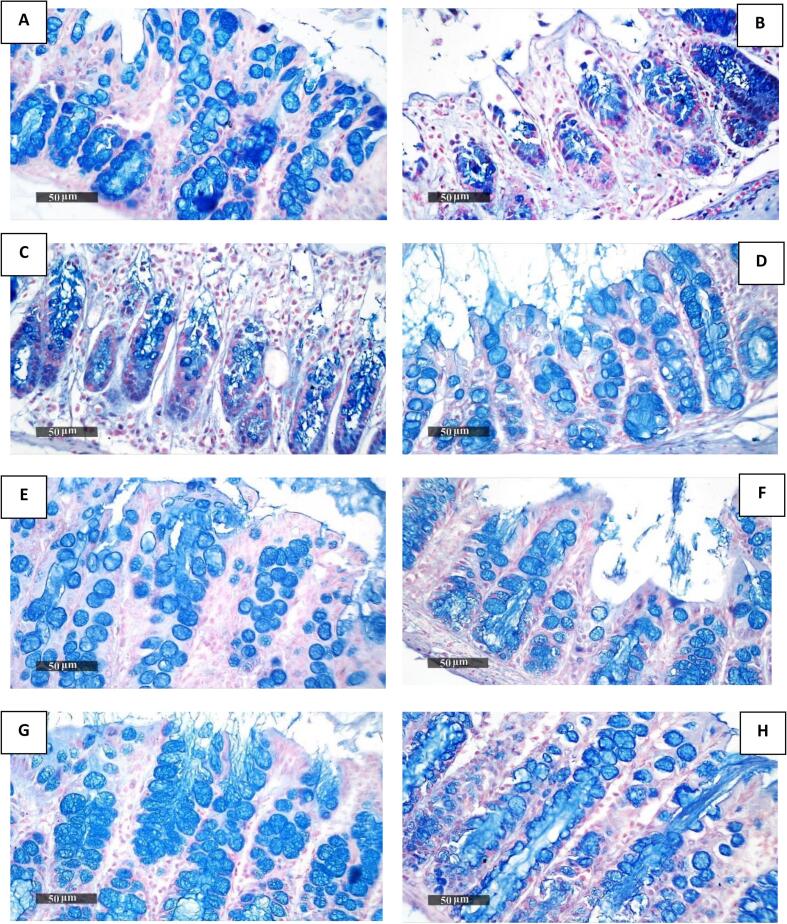
Photomicrograph of colon tissue sections from all groups stained with alcian blue. **A** from Group I demonstrates high expression of goblet cells' acidic mucin levels. Both Group II (B) and Group III (C) display a decrease in goblet cells' acidic mucin levels. The remaining Groups IV-VIII demonstrate an increase in goblet cells' acidic mucin levels (D–H). (For interpretation of the references to colour in this figure legend, the reader is referred to the web version of this article.)

Quantitative analysis of the colonic tissue sections revealed significantly reduced levels of goblet cells' acidic mucins in the colons from Group II (colitis model) (Fig. 4B) and Group III (Fig. 4C) relative to Group I (normal model) (Fig. 4A). Conversely, a significant increase in goblet cells' acidic mucin levels was recorded in Groups IV, V, VII, and VIII (Figs. 4D-H) relative to Groups II (Fig. 4B) and III (Fig. 4C). The maximum increase was observed in the group that received adult worm antigen as both a prophylactic and therapeutic (VIII) (Fig. 4H) (Table 3).

**Table 3:** The percent area of reactive goblet cell mucin using alcian blue in colonic tissues in different groups.

Groups	Area% of reactive goblet cells mucin	
	Mean ± SE	ANOVA *p*-value
I	35.3 ± 0.7	0.001**
II	11.3 ± 0.7	
III	15.6 ± 0.6	
IV	25.9 ± 0.5	
V	24.6 ± 1	
VI	26.5 ± 0.8	
VII	30 ± 0.8	
VIII	33.1 ± 0.9	

### 3.3 Immunohistochemical findings

#### 3.3.1 The expression of iNOS in colonic tissue of different experimental groups

**Fig. 5:**
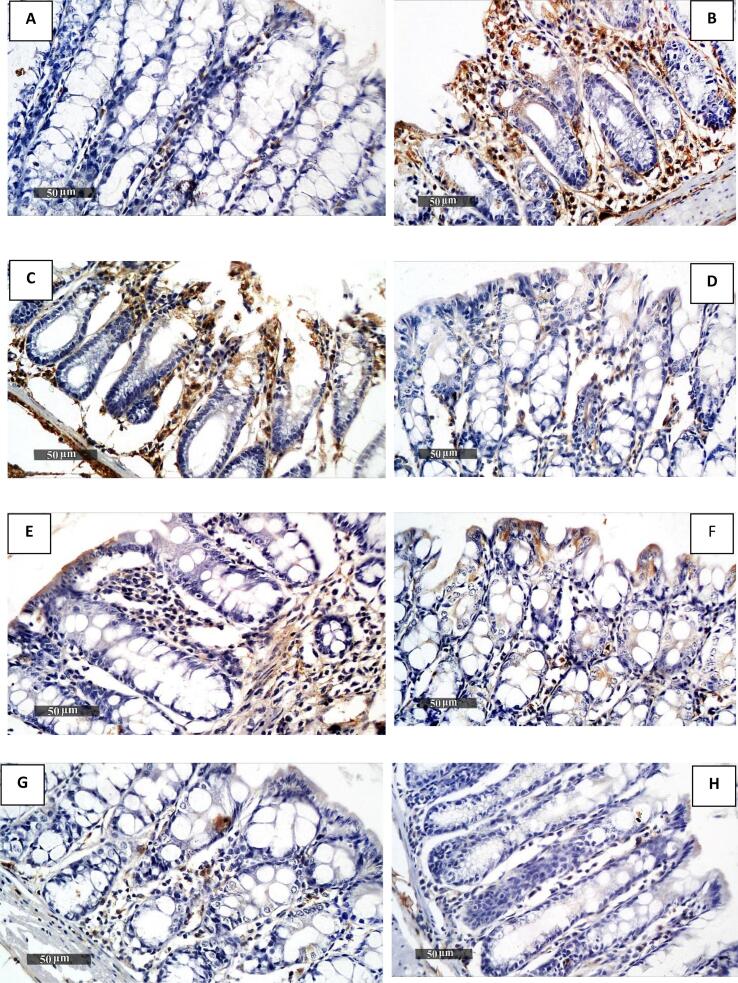
Immunohistochemical staining of colonic iNOS expression in all groups (A–H). The normal model Group I (A) showed the least amount of iNOS expression in colonic sections; the colitis model Group II (B) exhibited the most expression; Group III (C) showed increased expression; and Groups IV to VIII (D–H) demonstrated decreased expression.

**Fig. 6:**
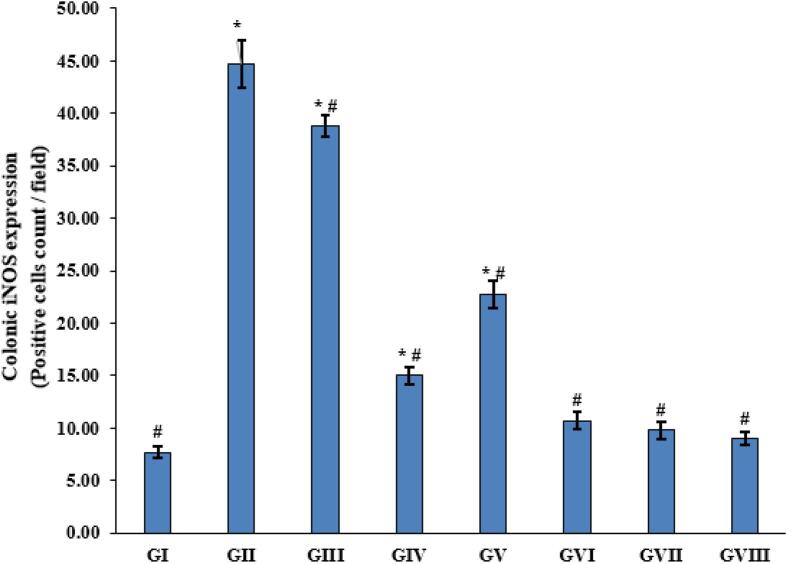
Statistical analysis of the results as positive cell count per field. The graph's data are presented as mean ± SE; *p < 0.05 compared to Group I of the normal model, and *#p* < 0.05 compared to Group II of the colitis model.

The effect of *T. spiralis* muscle larval antigen and adult worm antigen administration on colonic iNOS expression is presented in Fig. 5, Fig. 6. Colonic sections immunostained for iNOS revealed minimal brown staining in the normal model Group I (Fig. 5A) and positive brown staining in the colitis model Group II (Fig. 5B). An increase in positive brown staining was detected in the third group that received muscle larvae antigen as a prophylactic only (Fig. 5C). Relative to the colitis model Group II (Fig. 5B), the remaining Groups (IV, V, VI, VII, and VIII) (Figs. 5D-H) exhibited a notable decline in positive brown staining.

#### 3.3.2 TNF-α expression in colonic tissue of different experimental groups

**Fig. 7:**
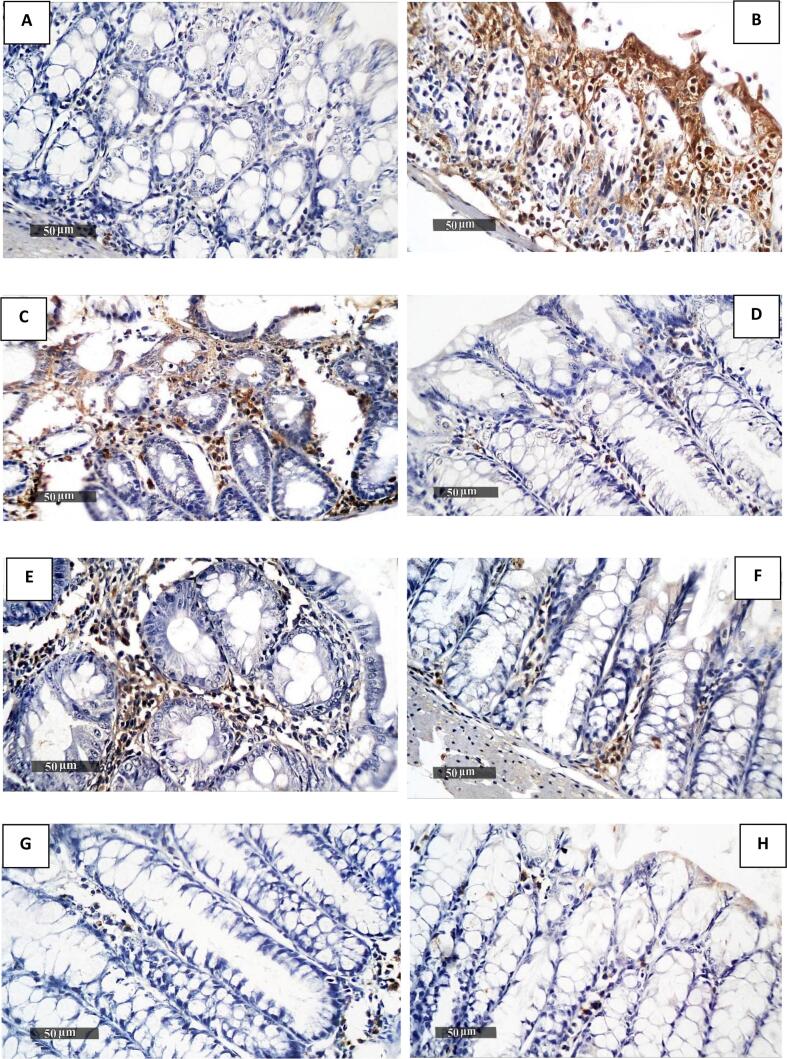
Immunohistochemical staining of colonic TNF-α expression in all groups (A–H). Colonic sections from the normal model Group I (A) showed the least TNF-α expression; Group II (B) exhibited the most expression; Group III (C) demonstrated increased expression; and Groups IV to VIII (D–H) displayed decreased expression.

**Fig. 8:**
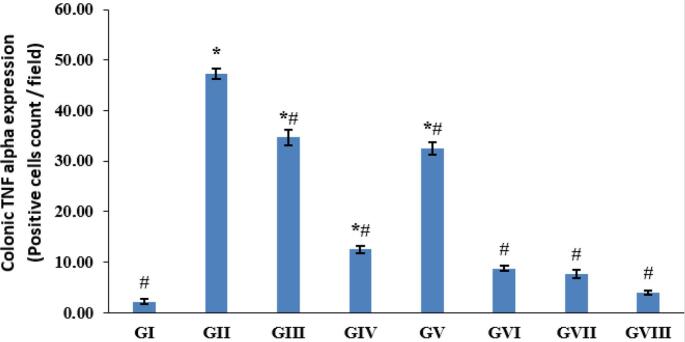
Statistical analysis of the positive cell count per field. The data are presented as mean ± SE. *p < 0.05 vs. Group I of the normal model, and *#p* < 0.05 compared to Group II of the colitis model.

The impact of *T. spiralis* muscle larval antigen and adult worm antigen administration on colonic TNF-α expression is presented in Fig. 6, Fig. 7. Colonic sections immunostained against TNF-α revealed minimal brown staining in the normal model Group I (Fig. 6A) and positive brown staining in the colitis model Group II (Fig. 6B). An increase in positive brown staining was detected in the third group that received muscle larvae antigen as a prophylactic only (Fig. 6C). As shown in Figs. 6D-H, compared to the colitis model Group II (Fig. 6B), a significant decline in positive brown staining was recorded in Groups IV, V, VI, VII, and VIII. (See Fig. 8.)

### 3.4 IFN-γ and IL-10 cytokine serum levels

**Fig. 9:**
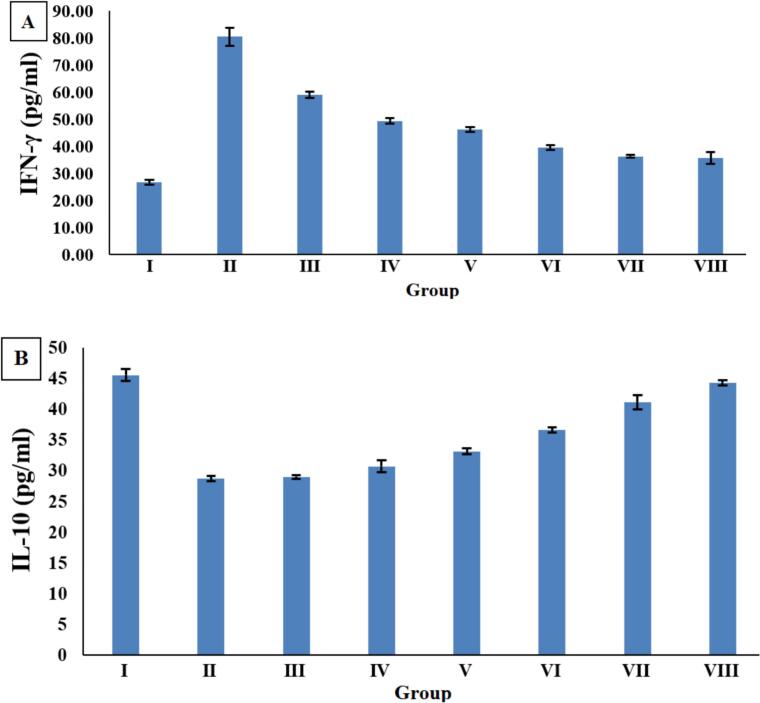
A: IFN-γ level; B: IL-10 level, in serum of different experimental groups.

Mice in colitis-induced Group II displayed a significant increase (*p* < 0.005) in IFN-γ levels (80.44 ± 3.32) compared with normal mice in Group I (26.63 ± 0.88). All treatment regimens normalized IFN-γ levels significantly (*p* < 0.005); however, mice injected with adult antigen as both prophylaxis and treatment (Group VIII) showed the best results (35.68 ± 2.10), followed by mice exposed to larval antigen as prophylaxis and treatment (Group VII) (36.32 ± 0.42), and then the treated Group VI with adult antigen (39.53 ± 0.84). Mice subjected to injection with larval antigen as treatment (Group V) showed better improvement (46.20 ± 0.90) compared to Group IV, which received injection with adult antigen as prophylaxis (49.40 ± 0.95). Finally, the third group, which was subjected to prophylaxis with larval antigen, recorded a level of 59.10 ± 1.16 (Fig. 9A).

Our results indicated that mice in the colitis-induced Group II showed a significant decrease (*p* < 0.005) in IL-10 levels (28.66 ± 0.46) compared with the normal Group I (45.46 ± 0.98). All treatment regimens could normalize IL-10 levels significantly (*p* < 0.005); however, Group III, injected with adult antigen as both prophylaxis and treatment, showed the best results (44.26 ± 0.40), followed by Group VII, which was exposed to larval antigen for prophylaxis and treatment (41.03 ± 1.16), and then the sixth group treated with adult antigen (36.56 ± 0.46). Conversely, mice injected with larval antigen as treatment (Group V) showed better improvement (33.10 ± 0.51) compared to Group IV, which received adult antigen as prophylaxis (30.63 ± 0.98), followed by Group III that was exposed to prophylaxis with larval antigen (28.93 ± 0.28) (Fig. 9B).

## 4 Discussion

Numerous epidemiological and experimental studies have suggested that early exposure to pathogens can guarantee the appropriate development of immunoregulatory mechanisms and lower the risk of developing autoimmune diseases, despite evidence supporting the theory that infections can cause autoimmunity (Rook and Bloomfield, 2021). In modern, highly developed nations with excellent healthcare and sanitation, the immune system is isolated from certain organisms that helped shape it during coevolution. This isolation can lead to dysregulation in the immune system and the emergence of inflammatory diseases like inflammatory bowel disease (IBD) (Rook and Bloomfield, 2021).

Depending on the helminthic species, the makeup of their products, and the host organism, the impact of helminthic infections or their byproducts on the emergence of various inflammatory diseases varies (Radovic et al., 2015). Due to the potential hazards associated with live helminthic treatment, research has concentrated on finding helminth-derived compounds that can modulate the immune system and might be administered to patients safely (Harnett and Harnett, 2010). The effects of these compounds are being thoroughly studied because, when administered in the appropriate amount, time, and route, they may produce immunomodulation strong enough to combat inflammatory diseases (Radovic et al., 2015).

It has previously been shown that helminthic infection positively impacts the course of several autoimmune disorders, including inflammatory bowel disease (Elliott and Weinstock, 2012). A *Trichinella spiralis* infection has demonstrated the potential to ameliorate the consequences of various autoimmune and allergic conditions, including airway disease, Type 1 diabetes, experimental autoimmune encephalomyelitis, and inflammatory bowel disease (Eissa et al., 2016).

In the current work, acetic acid was used to induce experimental colitis in mice, which aligns with many previous studies (Ali et al., 2018; Govindarasu et al., 2021; Ghasemi-Dehnoo et al., 2022). According to Gautam et al. (2013), the administration of intracolonic acetic acid resulted in increased mucoid/bloody diarrhea, inflammation, stool frequency, and injury to the colonic mucosa. Previous reports indicate that acetic acid causes ulcerative colitis in experimental mice (Das and Kanodia, 2012; Minaiyan et al., 2014). The main characteristics of ulcerative colitis include mucosal erosion, mucosal necrosis, ulceration, bleeding, and inflammation caused by protons released into the epithelium, leading to intracellular epithelial acidification that severely damages the epithelium.

Following previous observations related to disease activity index (DAI) (Gautam et al., 2013), our study showed that the second group (colitis model) exhibited a significant increase in DAI and the colon weight/length ratio, as evidenced by a decrease in body weight, along with the induction of diarrhea and severe bloody stools, compared to the healthy Group I. Additionally, we observed a significant reduction in the quantitative analysis of the colonic tissue sections for goblet cells' acidic mucin levels in the colon of the colitis model (Group II) and Group III, which received muscle larvae antigen as prophylaxis, compared with samples from the normal healthy Group I.

*Trichinella* antigens from muscle larvae, adult worms, and newborn larvae are stage-specific. Each stage of the *T. spiralis* life cycle generates unique antigens that can trigger the protective immunological responses of a particular host (Bruschi et al., 2022). In this context, the results of our study showed that the prophylactic administration of *T. spiralis* muscle larval antigens to mice before acetic acid-induced colitis (Group III) demonstrated a non-significant difference in disease activity index (DAI) compared to Group II. However, a very significant decrease in DAI was detected in Groups IV-VIII.

A marked decrease in DAI, with no significant difference (*p* < 0.05), was observed in Group VIII, which received adult *T. spiralis* antigens before and after the administration of acetic acid, correlating with the healthy mice (Group I) indicated by an improvement in body weight and resolution of clinical symptoms of colitis. Additionally, a significant increase in goblet cells' acidic mucin levels was recorded in Groups IV-VIII compared to Groups II and III. The maximum increase was seen in the group that received adult worm antigens as prophylactic and therapeutic treatment (Group VIII).

Our results documented a more significant improvement in groups that received adult antigen as prophylactic and therapeutic doses, which coincides with Motomura et al. (2009), who reported that the administration of *T. spiralis* antigens as prophylactic and therapeutic treatment induced better improvement in the form of decreased colitis severity. Similarly, our results align with those of Sun et al. (2019), who documented better improvement in airway inflammatory responses induced by ovalbumin when using adult antigens.

The results of the current study investigated the impact of *T. spiralis* muscle larvae antigen and adult worm antigen administration on colonic iNOS expression and TNF-α expression. Immunostained colonic sections for iNOS revealed minimal brown staining in the normal model group and increased positive brown staining in the colitis model group. Group III, which received muscle larvae antigen as prophylaxis only, showed non-significant results compared to the colitis model group. A significant decrease in positive brown staining was recorded in Groups IV-VIII relative to the colitis model Group II. These results align with previous observations that reported increased iNOS expression levels in colitis tissue samples and decreased expression in *T. spiralis* antigen-treated groups (Motomura et al., 2009; Gochman et al., 2012).

Research on various pathogen antigens has demonstrated that these antigens stimulate the host regulatory network, activating regulatory CD4+, CD25+, and Foxp3+ Tregs responsible for suppressing autoreactive effector T cells. This suppression, in turn, reduces the specific response against the parasite and the response towards autoantigens (McSorley et al., 2013).

The host regulatory network and the induced activation of regulatory mechanisms align with our results regarding serum levels of IFN-γ and IL-10 cytokines, which showed a significant increase (*p* < 0.005) in IFN-γ levels in the colitis-induced Group II compared to the normal mice Group I. All treatment regimens could normalize IFN-γ levels significantly (*p* < 0.005); however, mice injected with adult antigen as prophylaxis and treatment (Group VIII) showed the best results, followed by mice exposed to larval antigen as prophylaxis and treatment (Group VII), then the treated Group VI with adult antigen. Our results showed that Group II exhibited a significant decrease (*p* < 0.005) in IL-10 levels compared with the normal Group I. All treatment regimens could normalize IL-10 levels significantly (*p* < 0.005); however, Group III, injected with adult antigen as prophylaxis and treatment, showed the best results, followed by Group VII, which was exposed to larval antigen prophylaxis and treatment, and then the sixth group that was treated with adult antigen. On the other hand, Group V showed better improvement compared to Group IV, followed by Group III.

## 5 Conclusions

Our results demonstrate that *T. spiralis* antigens, particularly those from adult worms, have a protective and therapeutic effect on experimental colitis, with superior efficacy when administered both before and after colitis induction through inflammation reduction and immune response modulation. Thus, *T. spiralis* antigens could be utilized to improve disease outcomes and develop a novel treatment method for ulcerative colitis, an inflammatory bowel disease. Further studies based on molecular analysis are recommended to elucidate the different mechanisms involved. Additionally, to evaluate the long-term effects of the antigens, it is suggested that a chronic phase be developed by modifying dosages and cycles that allow for eventual relapsing and chronic forms of intestinal inflammation.

## Funding

This research work was funded by the Institutional Fund Project under grant no. (IFPIP: 850-142-1443). The authors gratefully acknowledge the technical and financial support provided by the 10.13039/501100002701Ministry of Education and 10.13039/501100004054King Abdulaziz University, DSR, Jeddah, Saudi Arabia.

## CRediT authorship contribution statement

**Majed H. Wakid:** Writing – review & editing, Supervision, Project administration, Methodology, Investigation, Funding acquisition, Data curation, Conceptualization. **Walaa A. El Kholy:** Writing – original draft, Methodology, Investigation, Data curation, Conceptualization. **Muslimah N. Alsulami:** Writing – original draft, Methodology, Investigation, Data curation, Conceptualization. **Eman S. El-Wakil:** Writing – original draft, Methodology, Investigation, Data curation, Conceptualization.

## Declaration of competing interest

The authors declare that they have no competing financial interests or personal relationships that could have appeared to influence the work reported in this paper.
